# Interneurons in the mouse visual thalamus maintain a high degree of retinal convergence throughout postnatal development

**DOI:** 10.1186/1749-8104-8-24

**Published:** 2013-12-21

**Authors:** Tania A Seabrook, Thomas E Krahe, Gubbi Govindaiah, William Guido

**Affiliations:** 1 Department of Anatomical Sciences and Neurobiology, University of Louisville, 511 South Floyd Street, Louisville, KY 40202, USA; 2 Department of Anatomy and Neurobiology, Virginia Commonwealth University Medical Center, Richmond, VA 23298, USA

**Keywords:** Dorsal lateral geniculate nucleus, Interneuron, Retinogeniculate pathway, Retinal convergence

## Abstract

**Background:**

The dorsal lateral geniculate nucleus (dLGN) of the mouse thalamus has emerged as a powerful experimental system for understanding the refinement of developing sensory connections. Interestingly, many of the basic tenets for such developmental remodeling (for example, pruning of connections to form precise sensory maps) fail to take into account a fundamental aspect of sensory organization, cell-type specific wiring. To date, studies have focused on thalamocortical relay neurons and little is known about the development of retinal connections onto the other principal cell type of dLGN, intrinsic interneurons. Here, we used a transgenic mouse line in which green fluorescent protein (GFP) is expressed within dLGN interneurons (GAD67-GFP), making it possible to visualize them in acutely prepared thalamic slices in order to examine their morphology and functional patterns of connectivity throughout postnatal life.

**Findings:**

GFP-expressing interneurons were evenly distributed throughout dLGN and had highly complex and widespread dendritic processes that often crossed eye-specific borders. Estimates of retinal convergence derived from excitatory postsynaptic potential (EPSP) amplitude by stimulus intensity plots revealed that unlike relay cells, interneurons recorded throughout the first 5 weeks of life, maintain a large number (approximately eight to ten) of retinal inputs.

**Conclusions:**

The lack of pruning onto interneurons suggests that the activity-dependent refinement of retinal connections in dLGN is cell-type specific. The high degree of retinal convergence onto interneurons may be necessary for these cells to provide both widespread and local forms of inhibition in dLGN.

## Findings

The dorsal lateral geniculate nucleus (dLGN) serves as the primary relay of visual information to cortex. Underlying the faithful relay of retinal signals are the precise patterns of connectivity between retinal ganglion cells (RGCs) and thalamocortical relay cells. Initially, projections from both eyes terminate diffusely in dLGN and single relay cells receive input from a dozen or so RGCs [1-3]. However, the retinogeniculate pathway undergoes a period of activity-dependent refinement in which projections from the two eyes segregate to form non-overlapping eye-specific domains and the number of functional retinal inputs onto relay cells is reduced to a few, to reflect the adult-like pattern of convergence [4]. Far less is known about the pattern of retinal convergence onto interneurons, the other principal cell type present in dLGN. These local-circuit neurons are involved in feedforward inhibition onto relay cells and play a role in contrast gain control, shaping receptive fields of relay cells, and altering the temporal precision of retinal inputs [5,6].

Typically, the study of retinal convergence has been performed in acute thalamic slice preparations where synaptic responses of dLGN cells are evoked by electrical stimulation of the optic tract. However, using this approach to assess retinal convergence onto interneurons is difficult since interneurons comprise a very small percentage of the total population of dLGN cells in mouse [2,7] and they are not readily distinguished under differential interference contrast (DIC) optics. Here we overcame these obstacles by using transgenic mice that express enhanced green fluorescent protein (GFP) in γ-aminobutyric acid (GABA)ergic interneurons (GAD67-GFP) [8]. Such cell-type specific visualization via GFP allowed us to readily target interneurons for *in vitro* recordings and test whether the age-related pruning of retinal inputs onto dLGN cells varies by cell type.

### Results

**Figure 1:**
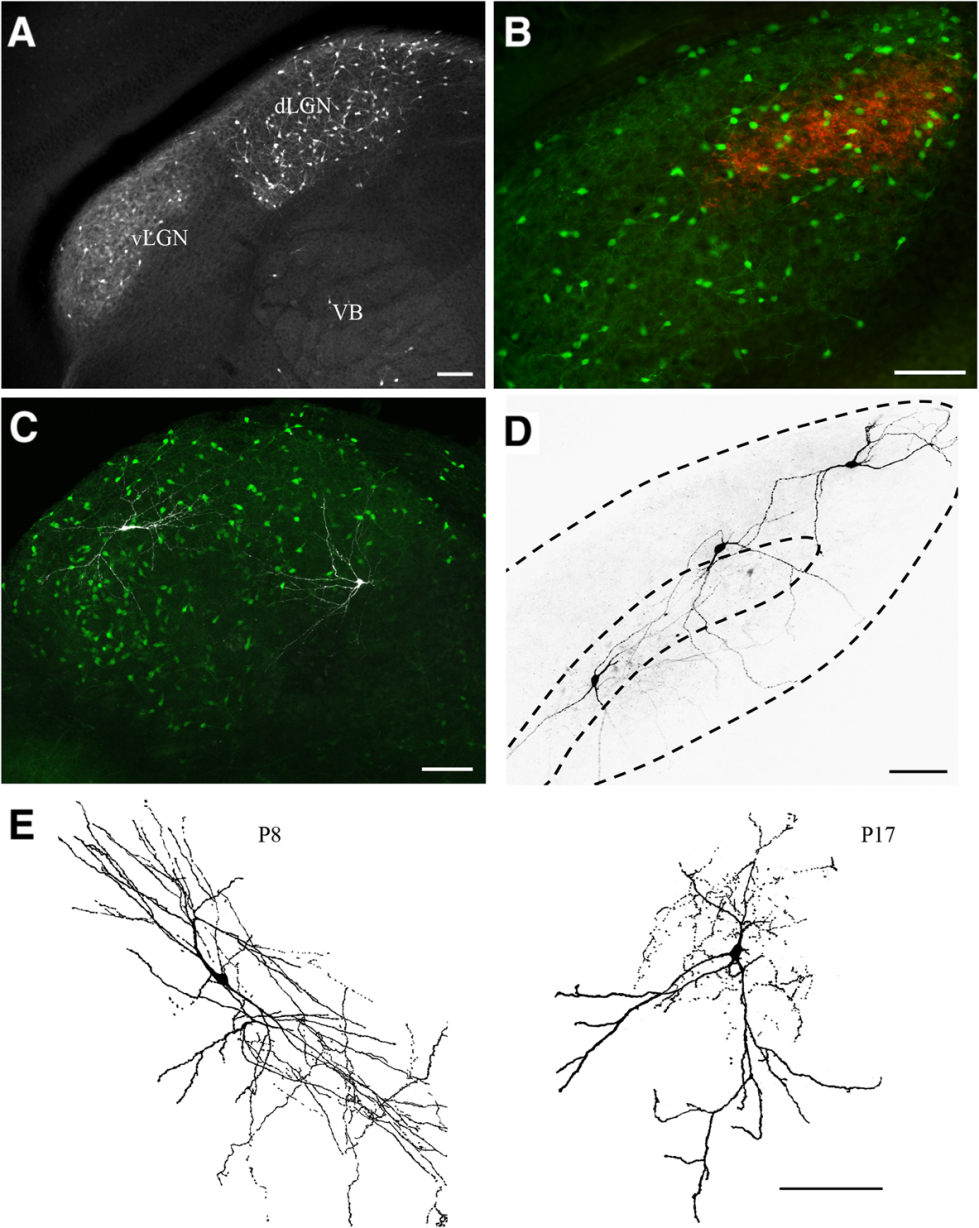
**Distribution of green fluorescent protein (GFP) expressing interneurons in dorsal lateral geniculate nucleus (dLGN). (A)** Coronal section of dLGN from a P7 GAD67-GFP mouse showing the distribution of GFP-expressing interneurons in thalamus (vLGN, ventral lateral geniculate nucleus; VB, ventrobasal complex). **(B)** Coronal section of dLGN from a P26 GAD67-GFP mouse that had one eye injected with cholera toxin subunit B (CTB) conjugated to Alexa Fluor 594. Interneurons in dLGN express GFP (green) and uncrossed retinogeniculate projections from the ipsilateral eye were anterogradely labeled with CTB (red). **(C)** Thalamic slice preparation at P11 showing z-stack projection image of GFP-expressing interneurons in dLGN labeled with biocytin during intracellular recordings. **(D)** Parasagittal section of P14 dLGN with biocytin labeled interneurons. Dashed lines outline the borders of dLGN and the region that corresponds to the region occupied by ipsilateral projections. **(E)** Z-stack projection images of GFP-expressing interneurons at P8 and P17 that were filled with biocytin during intracellular recording and then reconstructed using confocal microscopy (see Krahe *et al*. [10] for details). Scale bars, 100 μm. In all, 22 interneurons were labeled with biocytin (P7, n = 6 cells, 3 slices; P8, n = 1 cell, 1 slice; P10, n = 4 cells, 2 slices; P11, n = 2 cells, 1 slice; P14, n = 3 cells, 1 slice; P17, n = 3 cells, 3 slices; P20, n = 2 cells, 2 slices; P27, n = 1 cell, 1 slice).

In GAD67 mice, the expression of GFP in thalamus is restricted primarily to GABAergic interneurons intrinsic to dLGN and the lateral portion of the ventral lateral geniculate nucleus (Figure 1A). As expected, only a few interneurons were seen in the neighboring ventrobasal complex (Figure 1A) [7]. In this transgenic strain, GFP is not expressed in GABAergic cells of the thalamic reticular nucleus, therefore providing unambiguous visualization and access to intrinsic interneurons and their processes in dLGN [9]. From fixed tissue and 70 μm thick coronal sections through the middle of dLGN, the average density of GFP positive interneurons in dLGN was around one to two cells per 2500 μm^2^. At all ages examined (P7, P9, and P26), these cells were evenly distributed throughout dLGN (Figure 1B) and showed no preference for either the monocular or binocular regions of dLGN (one-way analysis of variance (ANOVA), *F* = 5.52, Bonferroni’s *post hoc* test, *P* >0.5 for all comparisons). However, biocytin labeling of individual interneurons during *in vitro* recordings showed that the branching pattern of dendrites was complex and expansive (Figure 1C). Indeed, processes of individual interneurons spanned large sectors of dLGN, sometimes even crossing eye-specific domains (Figure 1D).

**Figure 2:**
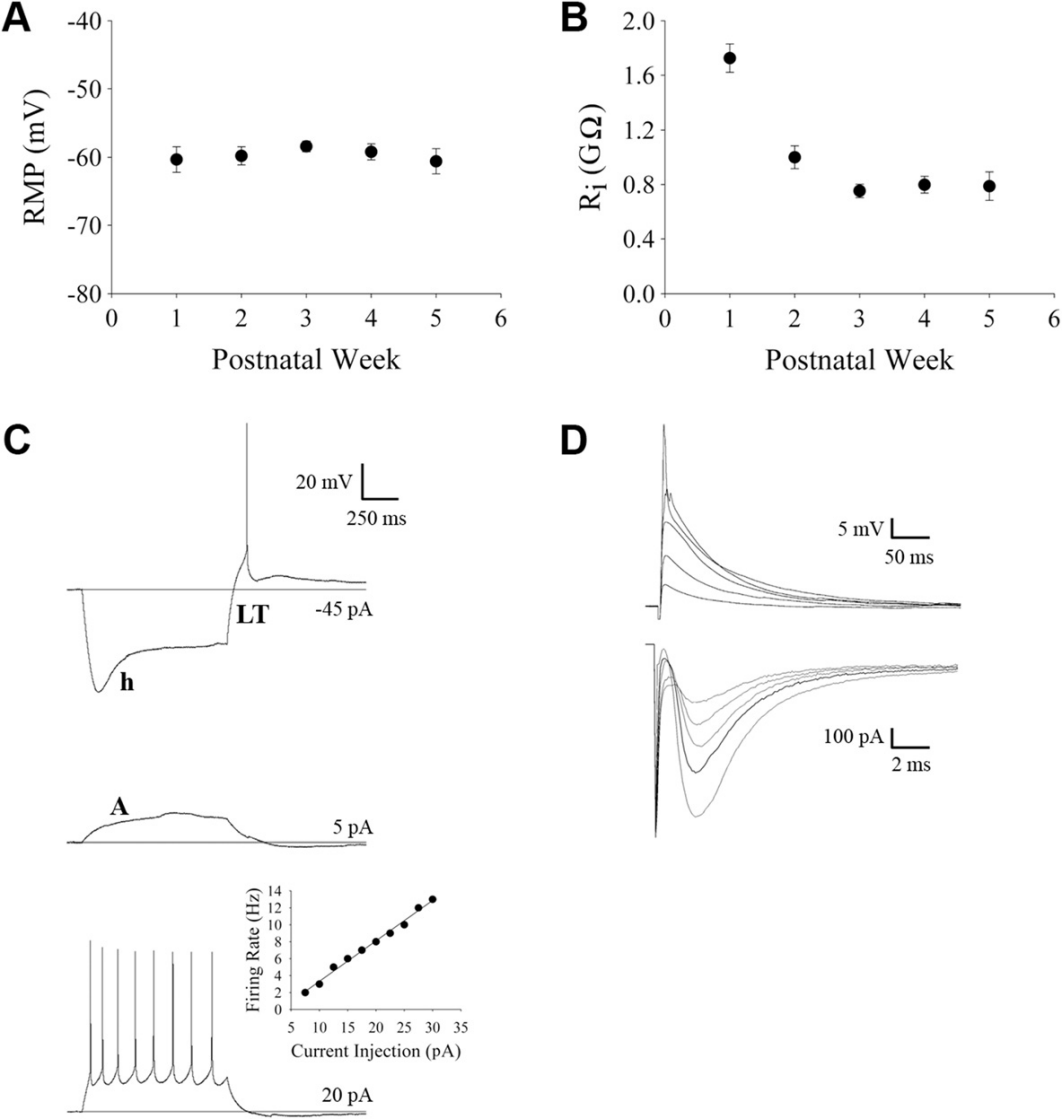
**Functional and morphological properties of green fluorescent protein (GFP) expressing interneurons in dorsal lateral geniculate nucleus (dLGN). (A,B)** Plots showing the resting membrane potential (RMP; (A)) and input resistance (R_i_; (B)) for a total of 72 interneurons recorded at different postnatal weeks (week 1, n = 6 cells, 1 slice; week 2, n = 15 cells, 10 slices; week 3, n = 27 cells, 14 slices; week 4, n = 14 cells, 4 slices; week 5, n = 10 cells, 2 slices). **(C)** Examples of voltage responses to varying current injection (indicated on the far right) at P10. The letters next to the traces correspond to some of the more salient active membrane properties of interneurons (h, hyperpolarization activated mixed cation conductance; LT, rebound low threshold Ca^2+^ spike; A, slow outward rectifying K^+^ conductance). The inset shows the linear relationship between spike frequency and current injection for responses in **(C)**. **(D)** Example of excitatory postsynaptic responses recorded at −65 mV in current (top, excitatory postsynaptic potentials (EPSPs)) or voltage clamp (bottom, excitatory postsynaptic currents (EPSCs)) of a P14 interneuron evoked by increasing levels of electrical stimulation (40, 45, 50, 55, and 60 μA).

Such specificity allowed us to readily identify and target dLGN interneurons for *in vitro* whole-cell recordings. Targeted recordings at different postnatal ages (P7 to P33, n = 72 cells) confirmed that GFP-expressing cells possessed the morphological (Figure 1C-E) and functional properties of rodent interneurons (Figure 2A-D) [11-17]. GFP-expressing cells labeled with biocytin (P7 to P27, n = 22 cells) exhibited type B morphology, having primary dendrites that originate from opposite poles of a small, spindle-shaped soma (Figure 1C-E). GFP-expressing cells also had a relatively high input resistance (R_i_) and more positive resting membrane potential (RMP) when compared to relay cells [14]. Similar to what others have shown in rat [17], we noted that RMP remained constant throughout the first 5 weeks postnatally (Figure 2A; one-way ANOVA, *F* = 0.56, Bonferroni’s *post hoc* test, *P* >0.5 for all comparisons), but showed a significant decrease in R_i_ between postnatal week 1 and all subsequent weeks examined (Figure 2B; one-way ANOVA, *F* = 9.34, Bonferroni’s *post hoc* test, *P* <0.001 for all comparisons with week 1). Voltage responses to depolarizing and hyperpolarizing square-wave current pulses were also consistent with those reported for interneurons (Figure 2C). Membrane hyperpolarization evoked a large depolarizing sag in the voltage response (h; Figure 2C, top). Termination of the hyperpolarizing current often led to the activation of a small rebound low threshold Ca^2+^ spike with an action potential riding its peak (LT; Figure 2C, top). Moderate depolarization led to tonic spike firing, but in a number of cells it also produced an outward rectification that delayed firing (A; Figure 2C, middle). Strong membrane depolarization evoked a high frequency train of spikes that displayed little if any frequency accommodation (Figure 2C, bottom).

**Figure 3:**
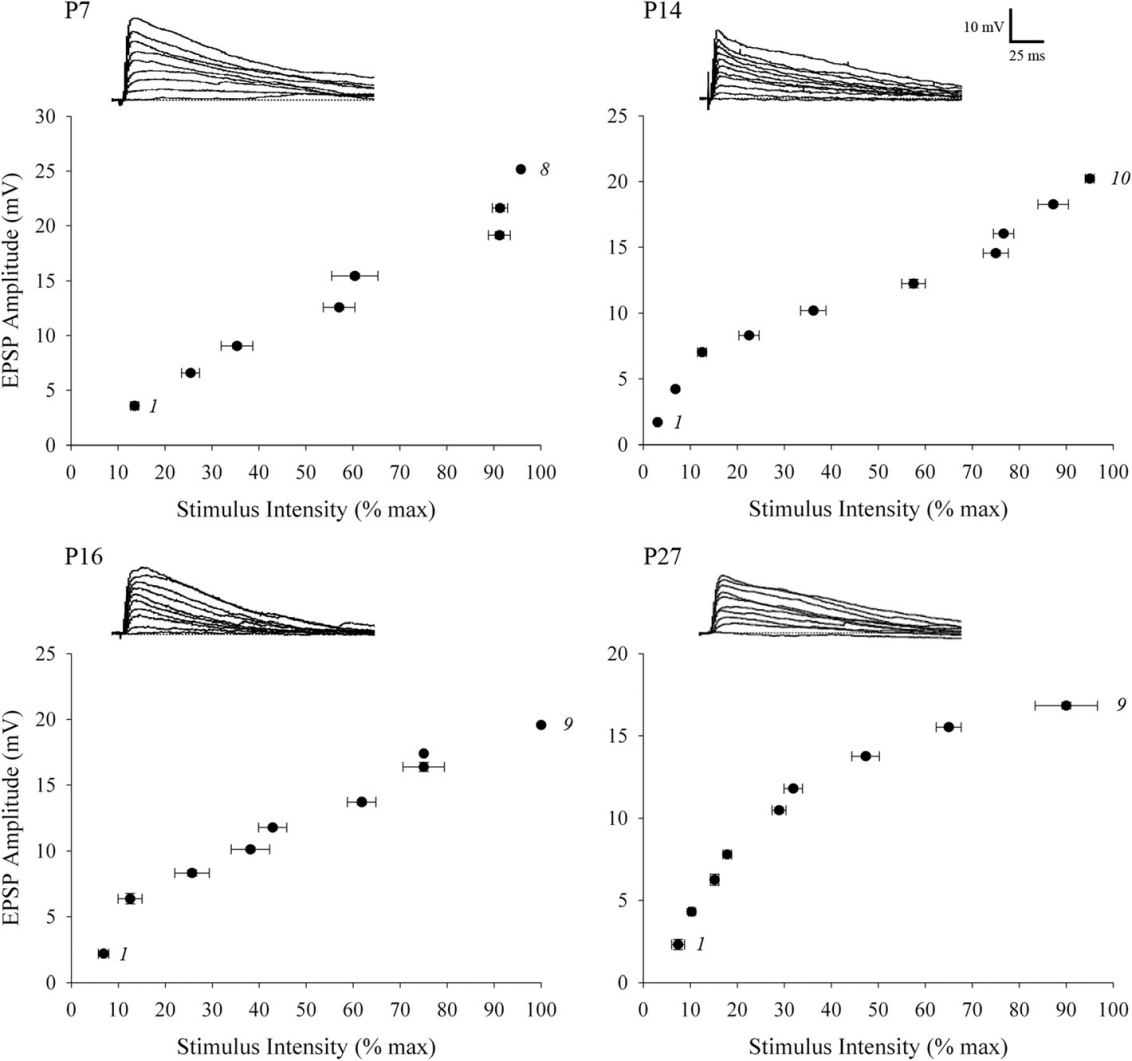
**Estimates of retinal convergence onto interneurons in dorsal lateral geniculate nucleus (dLGN).** Examples of synaptic responses evoked by progressive increases in the intensity of optic tract stimulation at P7, P14, P16, and P27. Corresponding excitatory postsynaptic potential (EPSP) amplitude by stimulus intensity plots are below each set of responses. Each point on the graphs depicts the means and SEMs for EPSP amplitude and stimulus intensity for a given retinal input. The interval that delineates one input from another was based on the value that corresponded to the single fiber response. Each graph was based on 66 to 122 responses. First and last recruited inputs are numbered. All responses recorded around −65 mV.

**Figure 4:**
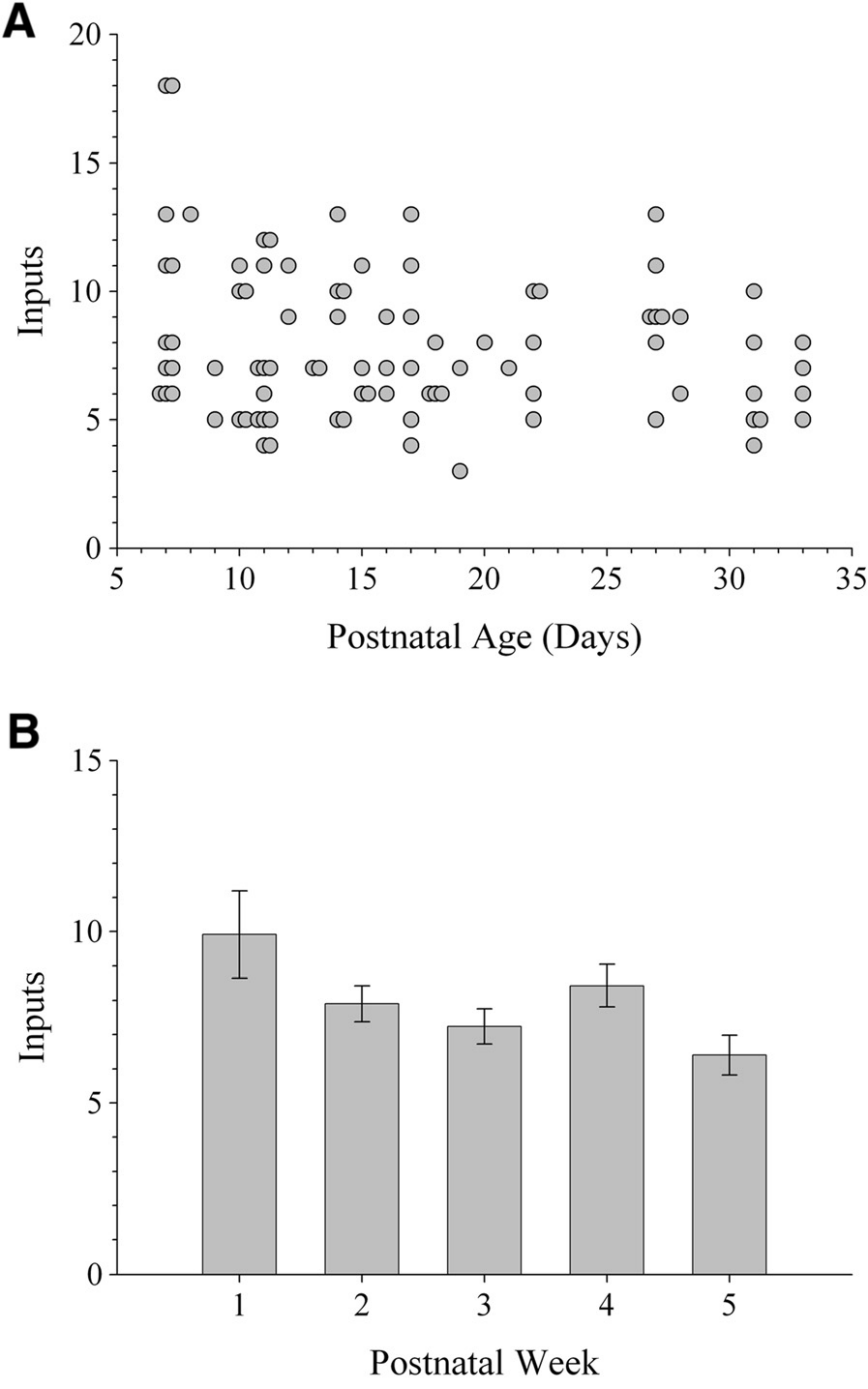
**Retinal convergence onto dorsal lateral geniculate nucleus (dLGN) interneurons during postnatal development. (A)** Scatterplot depicting the estimated number of retinal inputs as a function of postnatal day. Each point represents a single cell (n = 87 cells). **(B)** Summary plot showing the mean number of retinal inputs as a function of postnatal week. Error bars represent SEM (week 1, n = 12 cells; week 2, n = 30; week 3, n = 21; week 4, n = 14; week 5, n = 10).

To study the synaptic responses of interneurons and obtain estimates of retinal convergence, we recorded the postsynaptic activity (n = 7,730 excitatory postsynaptic potentials (EPSPs)) evoked by electrical stimulation of optic tract from a total of 87 identified interneurons between P7 to P33. For each cell we measured the amplitude of EPSPs evoked by progressive increases in stimulus intensity [1-3]. Single fiber responses, which were based on the minimal stimulus intensity needed to evoke a reliable response [3], were small (approximately 2 mV) and the amplitude at week 1 was not different from weeks 3 to 5 (one-way ANOVA, *F* = 3.77, Tamhane’s *post hoc* test, *P* >0.4 for all comparisons). At all ages, a progressive increase in stimulus intensity resulted in a graded increase in EPSP/excitatory postsynaptic current (EPSC) amplitude (Figures 2D and 3). Such response profiles reflect a high degree of retinal convergence [1-3]. In order to obtain estimates of retinal convergence we generated EPSP amplitude by stimulus intensity plots and adopted a criteria that was related to the amplitude of the single fiber response (see Methods for details). Representative examples shown in Figure 3 revealed that even at late postnatal ages interneurons receive as many as nine to ten retinal inputs. These input/output relations are summarized in Figure 4A, which plots estimates of retinal convergence for individual interneurons as a function of postnatal day. Throughout postnatal development interneurons received a high number of retinal inputs (approximately 8) with some receiving up to 10 as late as P31. When individual data were analyzed by postnatal week, the average number of inputs onto interneurons did not show a significant change with age (Figure 4B; one-way ANOVA, *F* = 2.60, Tamhane’s *post hoc* test, *P* >0.2 for all comparisons; week 1, n = 12 cells; week 2, n = 30; week 3, n = 21; week 4, n = 14; week 5, n = 10).

### Discussion

Using the GAD67-GFP mouse we were able to readily identify and target dLGN interneurons across a wide range of postnatal ages. We found that these GFP-expressing neurons were distributed evenly throughout dLGN and possessed the hallmark structural and functional features reported for rodent interneurons [11-17]. Most importantly, our results revealed that dLGN interneurons in mouse maintain a high level of retinal convergence throughout postnatal development. Even after 4 weeks of age, a single interneuron receives input from as many as eight to ten RGCs. This is in stark contrast to the pattern of convergence for developing relay cells which experience a fourfold to sixfold decrease, so by 3 weeks of age a single relay neuron receives input from only one to three RGCs [1-3]. It is important to note that our quantification of the number of retinal inputs a given interneuron receives is an estimate of retinal convergence. Indeed, our estimates may be influenced by such non-synaptic factors as ionic driving force and/or the activation of voltage-gated conductances. While such non-linearities could affect EPSP amplitude their potential impact would be similar across cells. We also acknowledge that there are a number of ways to assess retinal convergence and at least for the published studies pertaining to dLGN relay cells, they yield similar estimates [1-3]. Of notable significance, is that the EPSP amplitude by stimulus intensity plots of interneurons increased in a graded manner. A graded function reflects a high level of convergence, whereas a step-like one, a low level of convergence [1-3]. Interneurons maintain a graded function throughout development while relay cells show a change with age, from graded to step-like, suggesting retinal inputs onto relay cells are pruned during early postnatal life.

Such differences in the adult pattern of convergence are consistent with some of the known functional and structural features of these cell types. Compared to relay cells, interneurons have larger receptive fields [18-20] and tend to have a disproportionately higher number of retinal synapses compared to non-retinal ones [21,22]. Unlike relay cells, which provide the primary excitatory drive for visual cortical neurons, interneurons inhibit the activity of relay cells through complex synaptic arrangements that involve both conventional axonal (F1) as well as dendritic (F2) terminals [5,23]. Global inhibition encompasses large sectors of dLGN and seems to require coordinated input from several RGCs converging onto a single interneuron [24]. Such activation is needed in order to engage both F1 and F2 terminals that are distributed throughout the extensive processes of a given interneuron. Additionally, a more local form of inhibition can be accomplished via the direct activation of an isolated dendritic F2 terminal that makes contact with a single relay cell [25,26]. In this context, a single interneuron could have hundreds of these elements dispersed throughout their dendritic fields [5,26] potentially receiving input from many RGCs. Such high levels of convergence are even more likely when one considers that interneurons have highly complex and expansive dendritic fields [13,15-17] that can even extend across eye-specific domains.

Perhaps the most remarkable aspect of these results is the apparent lack of age-related retinal pruning onto interneurons. It is widely believed that such refinement is mediated by spontaneous retinal activity [4,27]. In developing dLGN relay cells, retinal activity evokes large excitatory postsynaptic potentials that activate plateau-like depolarizations that are mediated by high threshold, L-type Ca^2+^ channels [2,3]. The Ca^2+^ influx through L-type channels has been linked to cAMP response element-binding protein (CREB)-related signaling cascades proven to be critical for the refinement of retinogeniculate projections into segregated eye-specific domains [28,29]. While interneurons are reported to have L-type Ca^2+^ activity [24], we failed to detect retinally evoked plateau potentials. Thus, an intriguing possibility that warrants further testing is whether these events are the candidate mechanisms responsible for cell-type specific refinement.

### Methods

#### Subjects

Experiments were performed on GAD67-GFP mice (JAX, stock no. 007677, Bar Harbor, ME, USA) ranging in age from postnatal day (P) 7 to 33. The GAD67-GFP founder line was on a pigmented background (C57BL/6 × CB6F1/J). All analyses conformed to National Institutes of Health (NIH) guidelines and protocols, approved by the University of Louisville and Virginia Commonwealth University Institutional Animal Care and Use Committees.

#### In vitro slice physiology and intracellular filling

To examine the synaptic responses evoked by optic tract stimulation, we adopted an acute thalamic slice preparation, which preserves retinal connections and intrinsic circuitry in dLGN [1-3,10,23]. Mice were deeply anesthetized with isoflurane vapors and decapitated. Individual (300 μm thick) sections were cut in the parasagittal plane using methods described elsewhere [1,3,23]. Sections containing dLGN were placed into a recording chamber and maintained at 32°C and perfused continuously at a rate of 2.0 ml/min with oxygenated artificial cerebrospinal fluid (ACSF; 124 mM NaCl, 2.5 mM KCl, 1.25 mM NaH_2_PO_4_, 2.0 mM MgSO_4_, 26 mM NaHCO_3_, 10 mM glucose, and 2 mM CaCl_2_ (saturated with 95% O_2_/5% CO_2_), pH 7.4).

*In vitro* recordings were performed in the whole-cell current-clamp configuration with the aid of DIC and fluorescence optics on a fixed-stage, visualized recording apparatus (Olympus, EX51WI, Shinjuku, Tokyo, JP). Patch electrodes (3 to 7 MΩ) made of borosilicate glass were filled with a solution containing: 140 mM K-gluconate, 10 mM hydroxyethyl piperazine-ethanesulfonic acid (HEPES), 0.3 mM NaCl, 2 mM MgATP, 0.1 mM NaGTP, pH 7.25. Neuronal activity was digitized (10 to 20 kHz) through an interface unit (National Instruments), acquired and stored directly on the computer, and analyzed by using commercial software (Strathclyde Electrophysiology Software, Whole Cell Analysis Program, WinWCP V3.8.2, Glasgow, Scotland, UK). In some cases, the membrane properties and firing characteristics of interneurons were examined by recording the voltage responses to intracellular injections of square-wave current pulses.

To evoke synaptic activity in dLGN, square-wave pulses (0.1 to 0.3 ms, 0.1 to 1 mA) were delivered once every 20 s through a pair of thin-gauge tungsten wires (0.5 MΩ) positioned in optic tract. Stimulating electrodes were connected to a stimulus isolation unit (World Precision Instruments, A360) that received input from a computer controlled, multichannel pulse generator (World Precision Instruments, PulseMaster A300, Sarasota, FL, USA). Estimates of retinal convergence were determined by EPSP amplitude by stimulus intensity plots [2,3]. These were constructed by first determining the minimum stimulus intensity needed to evoke a postsynaptic response. Once the single fiber response was determined, current intensity was increased in small increments (0.5 to 1.0 μA) until a response of maximal amplitude was consistently reached [3]. A change in amplitude that was equal to or exceeded the value that corresponded to the single fiber response was used to distinguish one input from another. For each intensity value a minimum of five responses were obtained. It is important to note that we saw no evidence of retinally evoked inhibition in our recordings (however, see [14,15]), nor did we see a change in resting membrane levels even when the highest stimulus intensities were used. To further verify this we compared our recordings performed in normal ACSF with some performed in the presence of 20 μM bicuculine and 10 μM 3-aminopropyl(diethoxymethyl)phosphinic acid (CGP) to block GABA_A_-mediated and GABA_B_-mediated activity. There was no significant difference in the number of retinal inputs between cells recorded in the presence or absence of these GABA blockers (*t* test, *P* >0.5; mean retinal inputs ± SEM; P11 normal ACSF, 8 ± 1 vs P11 ACSF with GABA antagonists, 7 ± 1; n = 6 cells for both groups).

During some of the recordings a 0.1% to 0.2% biocytin solution containing (in mM): 130 K-gluconate, 10 HEPES, 8 NaCl, 2 MgATP, 0.1 NaGTP, pH 7.25 was included in the patch pipette and neurons were filled by passing alternating positive and negative current pulses (± 0.5 nA, 200 ms) through the recording electrode. After recording, these slices were fixed overnight with 4% paraformaldehyde in 0.1 M phosphate buffered saline (PBS), pH 7.2 and then incubated for 24 h in a 0.1% solution of Alexa Fluor 647 conjugated to streptavidin (Invitrogen, Carlsbad, CA, USA) dissolved in PBS with 0.1% Triton X-100. Slices were washed with PBS and then mounted with ProLong Gold antifade reagent (Invitrogen).

#### Cell density measurements of interneurons in dLGN

The overall density of interneurons was determined by counting GFP positive cells within the boundaries of dLGN. These measurements were obtained from 2 to 3 sections corresponding to the middle of dLGN (n = 3 mice; P7, P9, and P26). We also examined whether interneurons showed a preference between binocular and monocular regions of dLGN. To accomplish this we made ipsilateral eye injections of cholera toxin subunit B (CTB) conjugated to Alexa Fluor 594 (Invitrogen) to label uncrossed retinal projections within dLGN [2]. The spatial extent of the ipsilateral patch was then used to delineate monocular and binocular segments of dLGN.

## Abbreviations

A: Slow outward rectifying K^+^ conductance; ACSF: Artificial cerebrospinal fluid; ANOVA: Analysis of variance; Ca2+: Calcium; CaCl2: Calcium chloride; cAMP: cyclic adenosine monophosphate; CGP: 3-aminopropyl(diethoxymethyl)phosphinic acid; CREB: cAMP response element-binding protein; CTB: Cholera toxin subunit B; DIC: Differential interference contrast; dLGN: dorsal lateral geniculate nucleus; EPSC: Excitatory postsynaptic current; EPSP: Excitatory postsynaptic potential; F: Flattened vesicle; GABA: γ-aminobutyric acid; GAD: Glutamate decarboxylase; GFP: Enhanced green fluorescent protein; h: Hyperpolarization activated mixed cation conductance; HEPES: Hydroxyethyl piperazine-ethanesulfonic acid; K+: Potassium; KCl: Potassium chloride; LT: Rebound low threshold Ca^2+^ spike; L-type: Long-lasting; MgATP: Adenosine 5′-triphosphate magnesium salt; NaGTP: guanosine 5′-triphosphate sodium salt hydrate; P: Postnatal day; PBS: Phosphate buffered saline; RGCs: Retinal ganglion cells; Ri: Input resistance; RMP: Resting membrane potential; SEM: Standard error of the mean; VB: Ventrobasal complex; vLGN: ventral lateral geniculate nucleus.

## Competing interests

The authors declare that they have no competing interests.

## Authors’ contributions

TAS, TEK, and GG contributed to electrophysiological recordings and intracellular filling. TAS contributed to the imaging and reconstruction of biocytin labeled interneurons. WG performed eye injections. TAS carried out the collection, preparation, and imaging of fluorescently labeled tissue and the cell density measurements. TAS, TEK, and WG analyzed the data and performed the statistical analyses. TAS and WG designed the research and drafted the manuscript. All authors read and approved the final manuscript.
